# Supplementary material for: The leaderless communication peptide (LCP) class of quorum-sensing peptides is broadly distributed among Firmicutes
Source: Nat Commun. 2023 Sep 23;14:5947. doi: 10.1038/s41467-023-41719-3 (PMC10518010; doi:10.1038/s41467-023-41719-3)
Supplement: Supplementary file 1 — Supplementary Information [file 41467_2023_41719_MOESM1_ESM.pdf]

Supplementary Figure 1

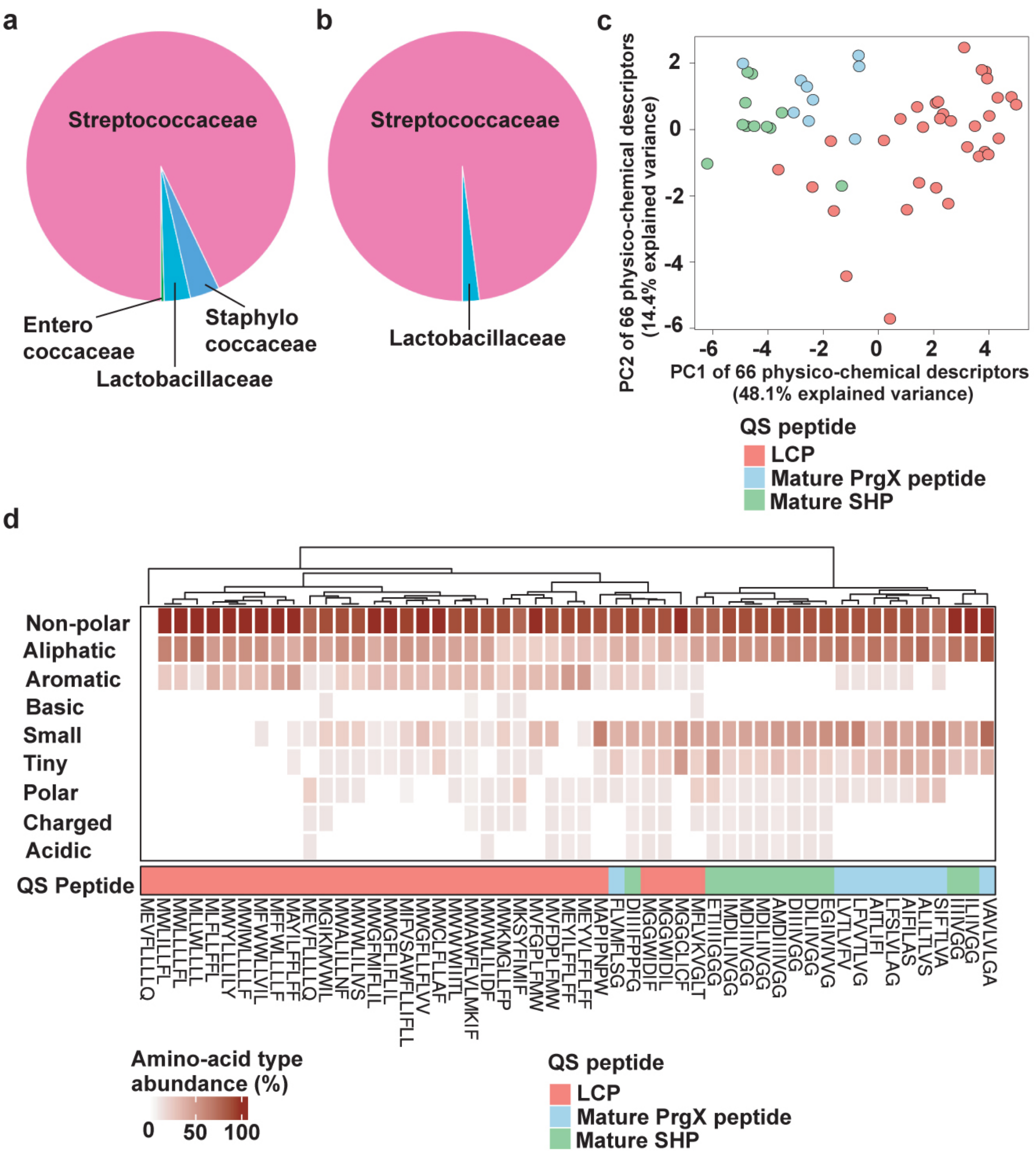

Supplementary Figure 1. The distribution of Rgg1-SHP (**a**) and Rgg2-SHP (**b**) systems in different bacterial taxa. **c**) Physico-chemical signatures of LCPs (red) and of PptAB-exported streptococcal SHPs (green) and enterococcal PrgX-like (blue) mature quorum sensing peptides. The panel displays the coordinates of each peptide sequence in the

two first principal components of the space of 66 physiochemical amino-acid descriptors (the 10 Blosum indices, 3 Cruciani properties, 6 FASGAI vectors, 10 Kidera factors, 3 MS-WHIM scores, 8 protFP descriptors, 8 ST-scales, 5 T-scales, 8 VHSE-scales and 5 Z-scales implemented in the ‘Peptides’ R package). **d**) Clustered heatmap representing the enrichment of each amino-acid type within LCPs (red), SHPs (green) and PrgX (blue): non-polar (A,C,F,G,I,L,M,P,V,W,Y), aromatic (F,H,W,Y), aliphatic (A,I,L,V), small (A,B,C,D,G,N,P,S,T,V), tiny (A,C,G,S,T), polar (D,E,H,K,N,Q,R,S,T,Z), charged (B,D,E,H,K,R,Z), acidic (B,D,E,Z) and basic (H,K,R).

Supplementary Figure 2

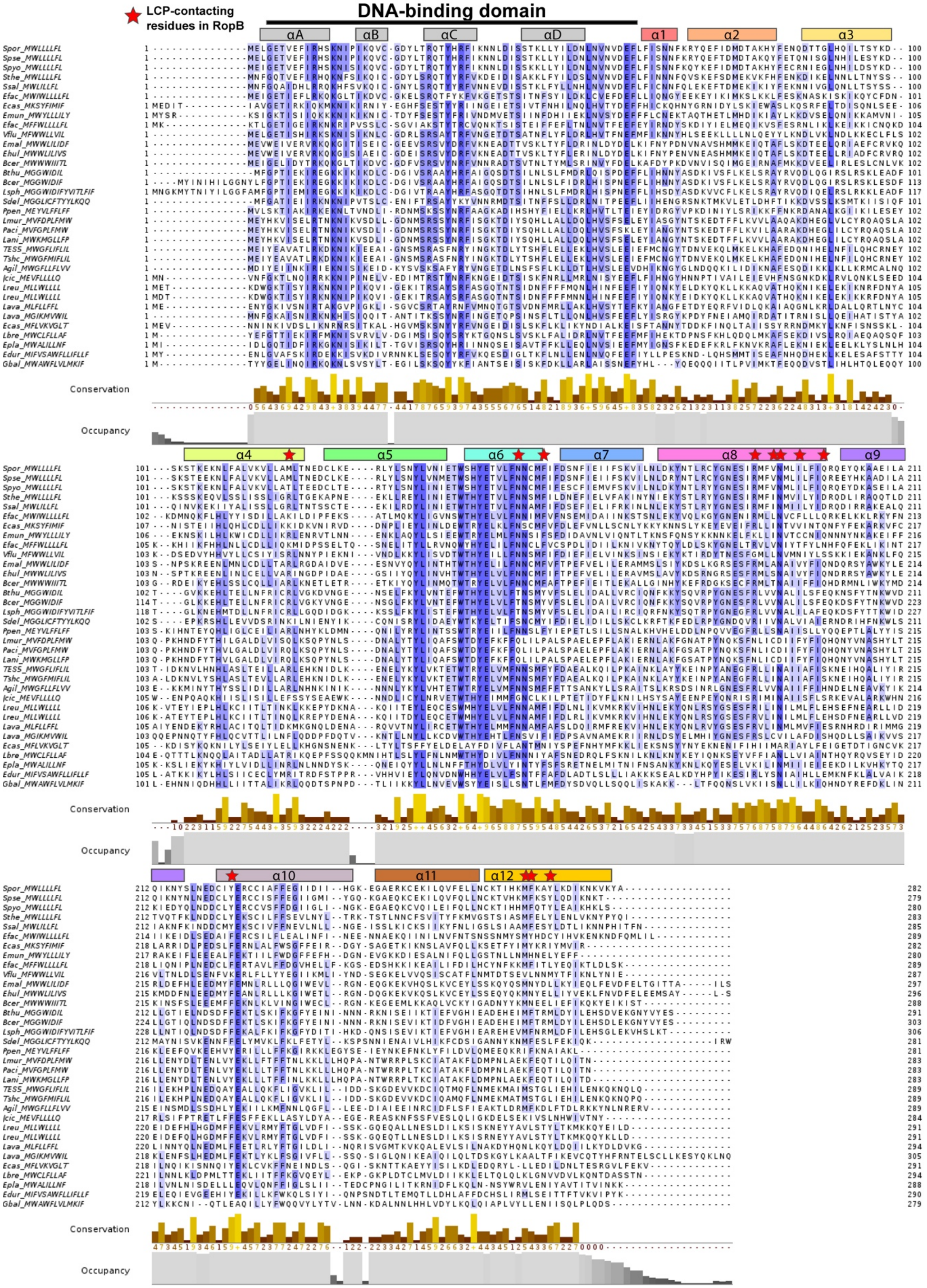

Supplementary Figure 2. Differential conservation of amino acids across regions of LCP clan receptors. Structure-guided multiple sequence alignment of receptors paired with LCPs. Each row depicts a receptor sequence, labeled according to their species origin and their cognate LCP. The structural domains and the LCP-contacting residues of RopB from *S. pyogenes* are mapped onto corresponding sites of the alignment. The conservation level of each site is displayed in the bottom of the alignment, and the most conserved amino acids at each site are highlighted in different shades of blue.

Supplementary Figure 3

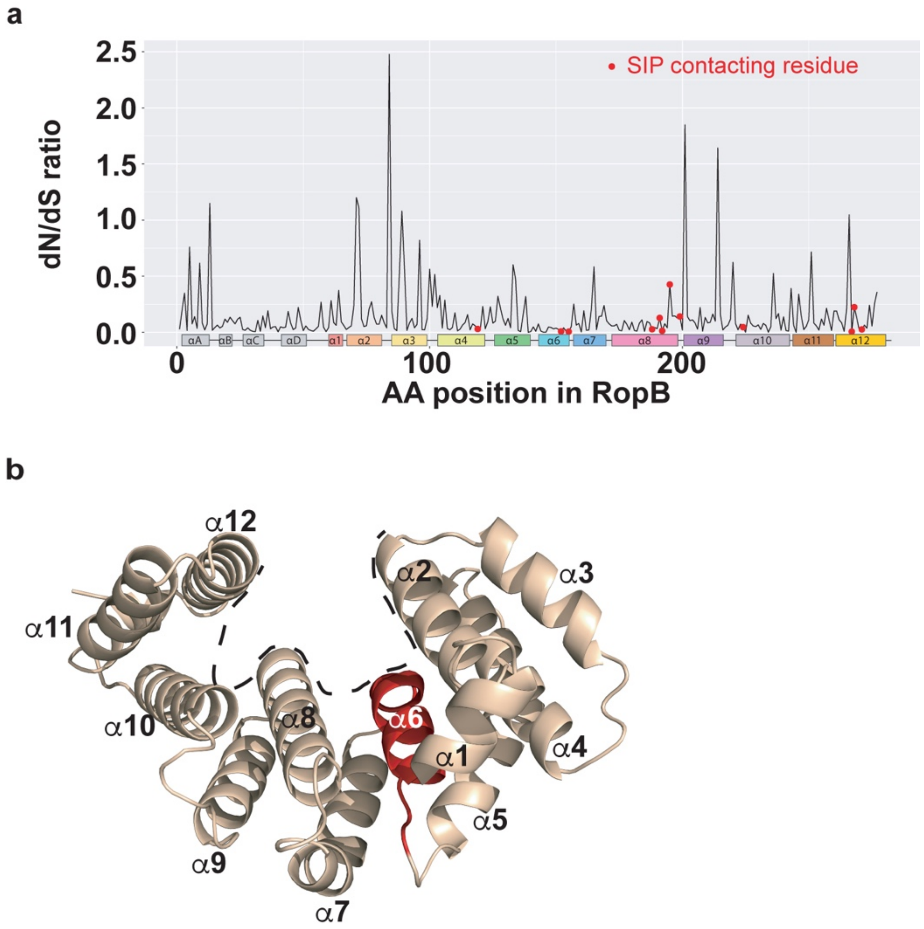

Supplementary Figure 3. Differential selective pressure across regions of RopB clan receptors. a, Site-wise dN/dS of each amino acid in the alignment in fig. S2 (the closer to 0, the higher the inferred purifying selection acting against amino acid substitution). Sites corresponding to LCP-contacting residues in RopB are highlighted in red. **b**, Structure of the LCP-binding pocket of RopB (PDB code: 6DQL). The boundary of the LCP-binding pocket is marked as dashed lines. The secondary structure (helices α1-12) of RopB-CTD is labeled and the highly conserved helix α6 is highlighted in red.

Supplementary Figure 4

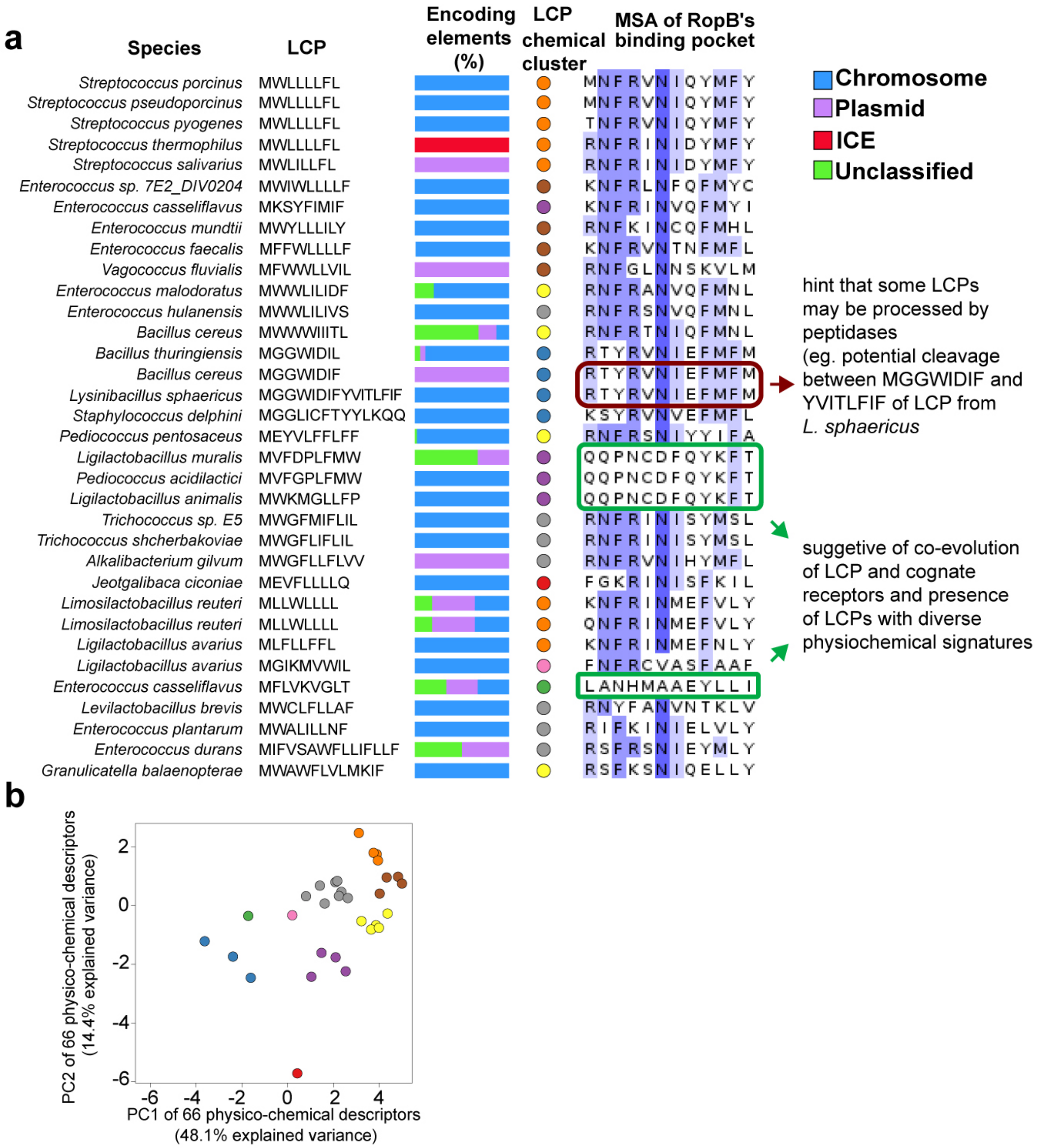

Supplementary Figure 4. Co-evolution receptor-LCP pairs. a, Mapping LCP-contacting residues and LCP sequences. Each line corresponds to a candidate receptor-LCP pair of a given species. The corresponding physiochemical cluster of the LCP is highlighted in color (as in panel b), and the LCP-contacting residues are displayed at the right. The LCP-contacting amino-acids are colored according to their conservation level

in the multiple sequence alignment. The green boxes indicate outlier LCP-binding residues, whose cognate LCPs also deviate from the canonical LCP signature in the PCA of peptide chemical descriptors. The red box highlights identical LCP-contacting residues associated with distinct LCPs, which nonetheless harbor an identical region. **b**, PCA of LCP chemical signatures.

Supplementary Figure 5

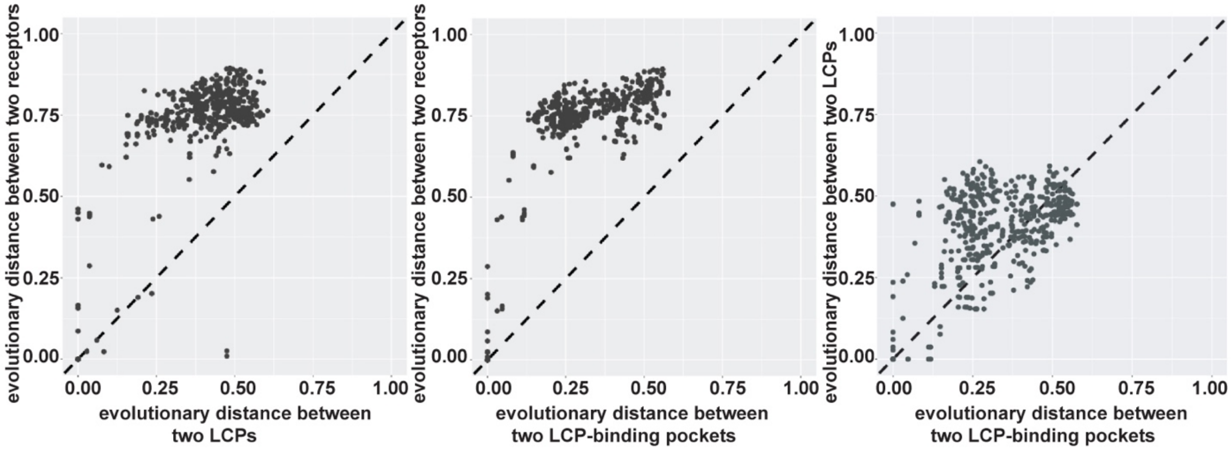

Supplementary Figure 5. Comparisons of pairwise evolutionary distances between entire receptors, LCP contacting residues, and predicted LCPs.

Supplementary Figure 6

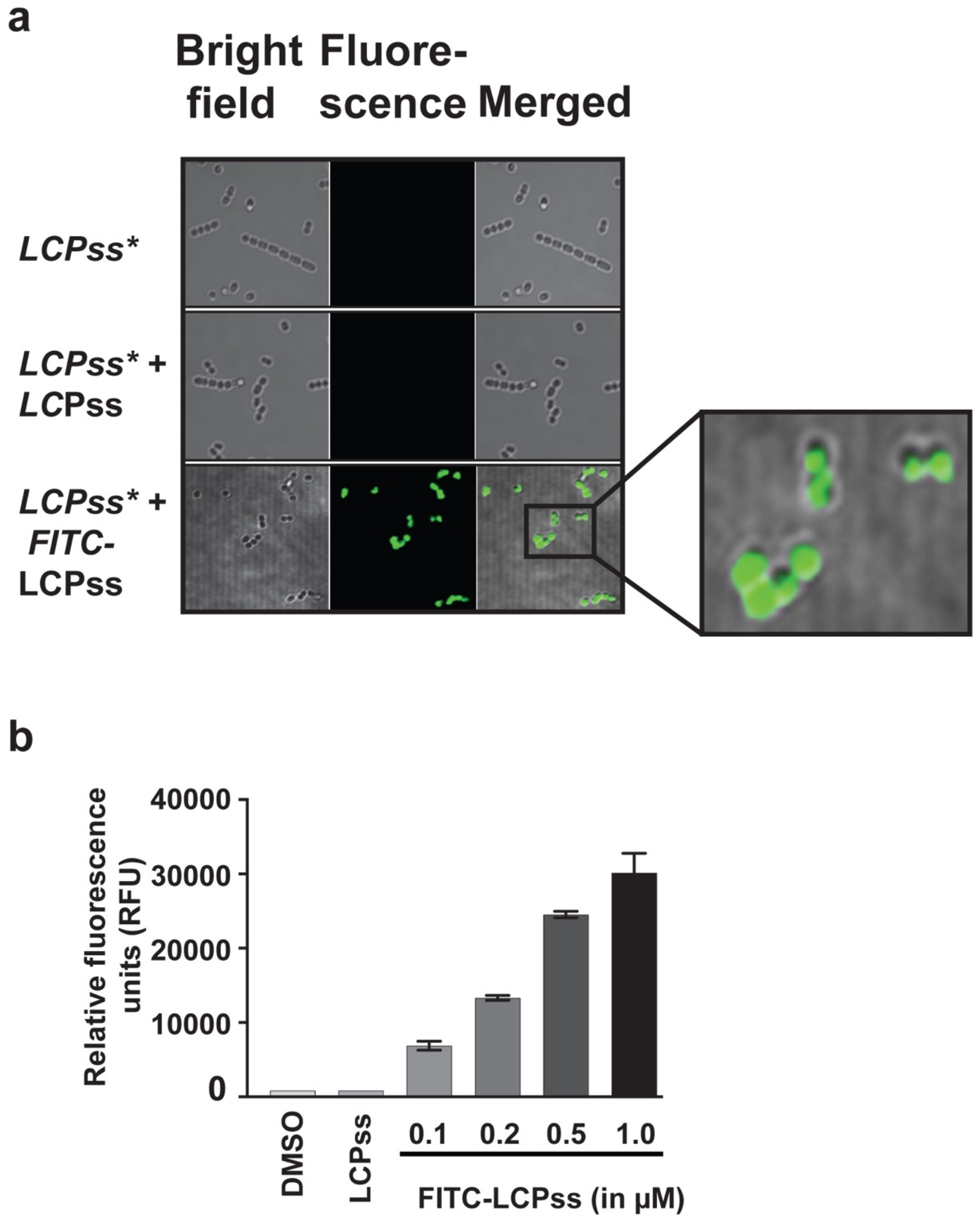

Supplementary Figure 6. a, Confocal microscopy images of the LCP_{ss}* mutant strain either unsupplemented or supplemented with the indicated synthetic peptide. For each sample, bright-field, fluorescence-field, and merged images are shown. Magnified view

of the FITC–LCP_{ss}–supplemented growth. **b,** The *LCP_{ss}** mutant strain was grown to the late exponential phase (A_{600} ~ 2.0) and supplemented with either the indicated synthetic peptide or the carrier for the synthetic peptides (DMSO). Unmodified LCP_{ss} was added at a final concentration of 1 μM, whereas varying concentrations FITC-LCP_{ss} were used. After 30 min of incubation at 37 °C, cells were washed three times with sterile PBS, suspended in PBS, and lysed. Fluorescence measurements were obtained with clarified cell lysates using excitation and emission wavelengths of 480 nm and 520 nm, respectively. The unsupplemented *LCP_{ss}** mutant strain was used as a reference, and changes in relative fluorescence units (RFU) relative to the reference are shown.

Supplementary Figure 7

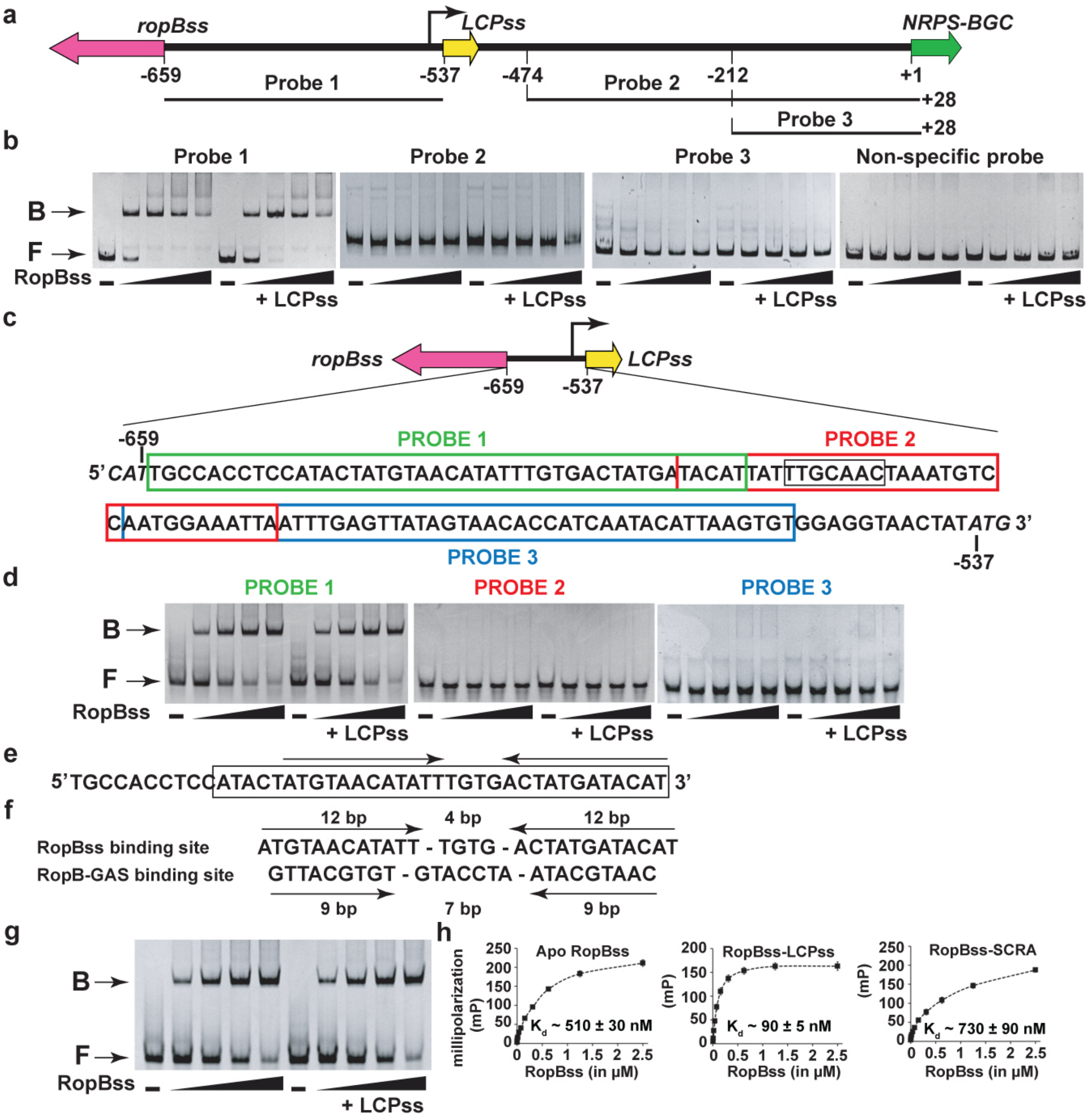

Supplementary Figure 7. Analysis of $RopB_{ss}$ binding sites within the *$ropB_{ss}$-NRPS-BGC* intergenic region. **a,** the *$ropB_{ss}$, LCP_{ss}*, and *NRPS-BGC* genes are marked by block arrows. The bent arrow above the line indicates the putative transcription start site of *LCP_{ss}* (*$PLCP_{ss}$*). Numbers indicate the nucleotide positions relative to the first nucleotide of *NRPS-BGC* start codon. Promoter fragments (probes 1-3) tested for $RopB_{ss}$ binding in

the electrophoretic mobility shift assay (EMSA) are shown as bars and the numbers at either side of each probe indicate the positions of 5' and 3' ends of the fragment relative to the first nucleotide of *NRPS-BGC* start codon. **b,** increasing concentrations of purified apo-$RopB_{ss}$ and LCP_{ss}-bound $RopB_{ss}$ (50, 100, 150, and 200 nM of $RopB_{ss}$) were incubated with the indicated probe and reaction mixtures were resolved on a 10% native-PAGE. Synthetic LCP_{ss} was added in the reaction mixture at a final concentration of 1 μM to generate LCP_{ss}-bound $RopB_{ss}$. The positions of free (F) and $RopB_{ss}$-bound (B) probes are marked. **c,** analysis of $RopB_{ss}$ binding sites within the *$ropB_{ss}$-LCP_{ss}* intergenic region. The nucleotide sequence within the *$ropB_{ss}$-LCP_{ss}* intergenic region is shown and the promoter fragments (probes 1-3) tested for $RopB_{ss}$ binding in the EMSA are boxed and shaded in different colors. **d,** increasing concentrations of purified apo-$RopB_{ss}$ and LCP_{ss}-bound $RopB_{ss}$ (50, 100, 150, and 200 nM of $RopB_{ss}$) were incubated with the indicated probe and reaction mixtures were resolved on a 10% native-PAGE. **e,** the nucleotide sequence of $RopB_{ss}$ binding site in *$PLCP_{ss}$* and the nucleotide sequence used in the FP studies is boxed. The inverted repeats within the $RopB_{ss}$ binding site are marked by arrows above. **f,** comparison of $RopB_{ss}$ and RopB-GAS binding sites. The 12-4-12 bp motif in $RopB_{ss}$ binding site and 9-7-9 bp motif in RopB-GAS binding site are labeled. **g,** increasing concentrations of purified apo-$RopB_{ss}$ and LCP_{ss}-bound $RopB_{ss}$ (50, 100, 150, and 200 nM of $RopB_{ss}$) were incubated oligoduplex containing the identified RopB-binding site and reaction mixtures were resolved on a 10% native-PAGE. **h,** Analyses of the binding between the FITC-labeled oligoduplex containing the putative $RopB_{ss}$-binding site and apo-$RopB_{ss}$, LCP_{ss}-bound, or SCRA-bound $RopB_{ss}$, as assessed by FP assay.

Supplementary Table 1. Bacterial strains and plasmids used in this study

**Strain or Plasmid**	**Description**	**Reference**
**Strains**		
WT *Streptococcus salivarius*	*Streptococcus salivarius* strain DS85_40B	https://www.ncbi.nlm.nih.gov/Traces/wgs/JAIQWT01?display=contigs
LCP_{ss}*	*S. salivarius* mutant strain that has the start codon of LCP_{ss} changed to stop codon in WT SAL	This study
LCP_{ss}*::LCP_{ss}	*S. salivarius* strain in which the stop codon introduced in the LCP_{ss}* mutant strain was reversed to start codon	This study
Δ$ropB_{ss}$	*S. salivarius* mutant strain that has the $ropB_{ss}$ coding region inactivated by marker less deletion in WT SAL	This study
Δ$ropB_{ss}$::$ropB_{ss}$	*S. salivarius* strain in which the $ropB_{ss}$ coding region was reintroduced in the Δ$ropB_{ss}$ mutant strain	This study
Enterococcus malodoratus	*Enterococcus malodoratus* DSM strain number DSM 20681	https://www.dsmz.de/collection/catalogue/details/culture/DSM-20681
Streptococcus porcinus	*Streptococcus porcinus* DSM strain number DSM 20725	https://www.dsmz.de/collection/catalogue/details/culture/DSM-20725
Limosilactobacillus reuteri	*Limosilactobacillus reuteri* DSM strain number DSM 32035	https://www.dsmz.de/collection/catalogue/details/culture/DSM-32035
E. coli DH5α	Host strain for cloning purposes	
E. coli BL21(DE3)	Host strain for protein overexpression, *F-, ompT, hsdSB(rB-mB-), gal (λ c I 857, ind1, Sam7, nin5, lacUV-T7 gene1), dcm(DE3)*	
**Plasmids**		

pJL	Low-copy number plasmid capable of replication in *Escherichia coli*, but a suicide vector in streptococcus. Chloramphenicol resistant. Used to generate isoallelic *S. salivarius* mutants	(*5*)
pET28a	Overexpression vector for N-terminally hexahistidine tagged recombinant proteins, KmR	Novagen
pET28a-ropB$_{ss}$	pET28a carries *ropB$_{ss}$* gene for overexpression of N-terminally hexahistidine tagged recombinant *ropB$_{ss}$*	This study

Supplementary table 2. Primers used in this study

Primer	Sequence 5' – 3'	Purpose
Δ*ropB*$_{ss}$ A	GTATCGATAAGCTTGATATCGAATTCCTGCAGCCCGGGGGATCTCCCATAGCTTTATTAAACGAAAATTTCTTTAC	5' primer for 5' region of *ropBss* to delete *ropBss*
Δ*ropB*$_{ss}$ B	GGATGAAATTAATGCATAAATGAAGATGATCGATTGCTTGTCC	3' primer for 5' region of *ropBss* to delete *ropBss*
Δ*ropB*$_{ss}$ C	GGACAAGCAATCGATCATCTTCATTTATGCATTAATTTCATCC	5' primer for 3' region of *ropBss* to delete *ropBss*
Δ*ropB*$_{ss}$ D	GGGATTTTGGTCATGAGATTATCAAAAGGATCGATATGTCAGGAGCCTATATGCCACTAG	3' primer for 3' region of *ropBss* to delete *ropBss*
LCP$_{ss}$* A	CAATACATTAAGTGTGGAGGTAACTATTAGTGGTTGATTTTACTATTTCTTTGA	5' primer of *LCPss* to change the *LCPss* start codon to stop codon
LCP$_{ss}$* B	TCAAAGAAATAGTAAAATCAACCACTAATAGTTACCTCCACACTTAATGTATTG	3' primer of *LCPss* to change the *LCPss* start codon to stop codon
Δ*oppAD*$_{ss}$ A	GTCAATTTACTTGGCAAAAGA	5' primer for 5' region of *oppA* to delete *oppADss*
Δ*oppAD*$_{ss}$ B	GTTATAGTTATTATAACATGTATTGATGGTTGTGTATAGA	3' primer for 5' region of *oppA* to delete *oppADss*
Δ*oppAD*$_{ss}$ C	TCTATACACAACCATCAATACATGTTATAATAACTATAAC	5' primer for *spc* along with *oppAss* overlap sequence to delete *oppADss*
Δ*oppAD*$_{ss}$ D	CATCCGGATGACAAGCCA	3' primer for 5' region of *oppD*ss to delete *oppADss*

Δ*oppAD*$_{ss}$ spcF	TCGCGAGCCGATAATATTACTTATAATTTTTTTAATCTGTTATTTAAATAG	5' primer for *spc* along with *oppDss* overlap sequence to delete *oppADss*
Δ*oppAD*$_{ss}$ spcR	CTATTTAAATAACAGATTAAAAAAATTATAAGTAATATTATCGGCTCGCGA	3' primer for *spc* along with *oppDss* overlap sequence to delete *oppADss*
*S. porcinus speB*_qRT Fwd	TGCGTGAAATTTGGTGAACAG	5' primer for *S. porcinus speB* qRT-PCR
*S. porcinus speB*_qRT Rev	ATATGGACTACGGCCCATCTA	3' primer for *S. porcinus speB* qRT-PCR
*S. porcinus tufA*_qRT Fwd	GGACACGCGGACTATGTTAAA	5' primer for *S. porcinus* reference gene *tufA* qRT-PCR
*S. porcinus tufA*_qRT Rev	AGGATGTGCTCACGAGTTTG	3' primer for *S. porcinus* reference gene *tufA* qRT-PCR
E. malodoratus 16S qRT Fwd	CATCCCTTGACGGTATCTAACC	5' primer for *E. malodoratus* reference gene *16S rRNA* qRT-PCR
E. malodoratus 16S qRT Rev	CTCGCTTTACGCCCAATAAATC	3' primer for *E. malodoratus* reference gene *16S rRNA* qRT-PCR
E. malodoratus T7SS qRT Fwd	ACGATGTGTGGGTCTCTTTATC	5' primer for *E. malodoratus T7SS* qRT-PCR

E. malodoratus T7SS qRT Rev	TCAACGAATCGTGCAGATGTA	3’ primer for *E. malodoratus T7SS* qRT-PCR
E. malodoratus T7-eff qRT Fwd	GTGACAAAGTCTCTGCCCAATA	5’ primer for *E. malodoratus T7-eff* qRT-PCR
E. malodoratus T7-eff qRT Rev	GAGGATCAAACTTCGCAGGTAA	3’ primer for *E. malodoratus T7-eff* qRT-PCR
E. malodoratus hypothetical *gene 1* qRT Fwd	CTAGCAGCTCTTCCATTTGTTTC	5’ primer for *E. malodoratus gene 1 rRNA* qRT-PCR
E. malodoratus hypothetical *gene 1* qRT Rev	CATAGAATTGACCTTGCGAATCG	3’ primer for *E. malodoratus gene 1 rRNA* qRT-PCR
L. reuteri tufA qRT Fwd	GAAGGTGACCCAGAACAAGAA	5’ primer for *L. reuteri tufA* qRT-PCR with the locus tag *A4V07_02000*
L. reuteri tufA qRT Rev	CATGAATGGCTTGTCAGTAGGA	3’ primer for *L. reuteri tufA* qRT-PCR with the locus tag *A4V07_02000*
L. reuteri ABC transporter *gene 1* qRT Fwd	CAAGTTGGTGTCGTCAGTGATA	5’ primer for *L. reuteri* ABC transporter *gene 1* qRT-PCR with the locus tag *A4V07_07970*
L. reuteri ABC transporter *gene 1* qRT Rev	CATAGAATTGACCTTGCGAATCG	3’ primer for *L. reuteri* ABC transporter *gene 1* qRT-PCR with the locus tag *A4V07_07970*
