# Supplementary material for: The leaderless communication peptide (LCP) class of quorum-sensing peptides is broadly distributed among Firmicutes
Source: Nat Commun. 2023 Sep 23;14:5947. doi: 10.1038/s41467-023-41719-3 (PMC10518010; doi:10.1038/s41467-023-41719-3)
Supplement: Supplementary file 3 — Description of Additional Supplementary Files [file 41467_2023_41719_MOESM3_ESM.pdf]

Description of Additional Supplementary Files:

Supplementary Data 1: Automated receptor-peptide mapping for all homologs of RopB. Tab 1: list of all flanking sORFs of all receptors, with indication of the cognate peptide chosen by our automated method. Tab 2: list of automatically-assigned receptor-QS peptide pairs, reduced to only representative receptors of groups of identical receptors.

Supplementary Data 2: Manually-refined receptor-peptide mapping in RopB and Rgg clans. List of receptors of the RopB, Rgg_I and Rgg_II clans, along with their manually chosen cognate peptide (if detected).
